# Sustainable Extraction and Multimodal Characterization of Fungal Chitosan from *Agaricus bisporus*

**DOI:** 10.3390/foods14162785

**Published:** 2025-08-11

**Authors:** Inês C. G. Sousa, Samiris C. Teixeira, Marinaldo V. de Souza, Maria B. M. Conde, Gabriela R. Bailon, Samuel H. S. Cardoso, Leandro D. Araújo, Eduardo B. de Oliveira, Sukarno O. Ferreira, Taíla V. de Oliveira, Nilda de F. F. Soares

**Affiliations:** 1 Department of Food Technology, Federal University of Viçosa, Campus Viçosa, Viçosa 36570-900, MG, Brazil; ines.sousa@ufv.br (I.C.G.S.); samiris.teixeira@ufv.br (S.C.T.); gabriela.bailon@ufv.br (G.R.B.); samuel.h.cardoso@ufv.br (S.H.S.C.); eduardo.basilio@ufv.br (E.B.d.O.); 2 Postgraduate Program in Materials Science (PPGCM), Federal University of Maranhao, São Luís 65080-805, MA, Brazil; marinaldo.vilar@discente.ufma.br; 3 Graduate Program in Food Technology (PPGTA), Department of Food, Federal University of Technology of Paraná, Curitiba 80230-910, PR, Brazil; mariabeatrizconde@alunos.utfpr.edu.br; 4 Department of Wine, Food and Molecular Bioscience, Lincoln University, Lincoln 7647, New Zealand; leandro.araujo@lincoln.ac.nz; 5 Department of Physics, Federal University of Viçosa, Campus Viçosa, Viçosa 36570-900, MG, Brazil; sukarno@ufv.br

**Keywords:** fungal chitosan, extraction, biopolymers

## Abstract

Biologically derived polymers, such as chitosan, have gained attention as sustainable alternatives to synthetic materials for food and biomedical applications. Fungal-derived chitosan offers notable advantages over crustacean-based chitosan, including a renewable origin and lower allergenic potential. In this study, chitosan was extracted from fungal biomass through a sequential process comprising demineralization, deproteinization, and deacetylation. The extracted material was characterized using Fourier transform infrared spectroscopy (FTIR), X-ray diffraction (XRD), thermogravimetric analysis (TGA), scanning electron microscopy (SEM), zeta potential measurement, dynamic light scattering (DLS), and color analysis. Compared to commercial chitosan, the fungal chitosan exhibited lower crystallinity and thermal stability, as well as a more porous surface morphology. Its degree of deacetylation and surface charge suggest promising potential for use in biodegradable films and functional materials. These findings highlight the feasibility of using fungal biomass as a sustainable and valuable source of chitosan for technological applications.

## 1. Introduction

In 1811, French researcher Henri Braconnot, while investigating fungi, was the first to isolate chitin, which he initially termed “fungine.” Subsequently, in 1823, another French scientist, Auguste Odier, identified a similar compound of animal origin extracted from insect exoskeletons. Odier named the substance “chitin,” a term that continues to be used today [1]. Chitin, one of the most abundant biopolymers in nature, is a polysaccharide that ranks second only to cellulose in terms of prevalence. It is primarily found in yeast, fungal cell walls, insects, and the exoskeletons of marine organisms. Despite its widespread availability, the use of chitin in the food industry remains limited due to several inherent challenges. Its semi-crystalline structure makes it rigid and difficult to process, and it also exhibits limited biodegradability and poor solubility in most organic solvents [2]. To overcome these limitations, chitin is commonly transformed into chitosan through a process known as deacetylation. During this chemical modification, acetyl groups are removed from the polymer backbone, resulting in structural changes that enhance its functional versatility. As a result, chitosan is more soluble than chitin, making it more suitable for a broad spectrum of industrial applications [3].

Due to its low toxicity, biocompatibility, and biodegradability, chitosan is a versatile biopolymer that is highly valued in the food, cosmetic, pharmaceutical, and agricultural industries [4]. Commercially, chitosan is typically extracted from crab exoskeletons; however, its production involves the use of harsh chemical reagents, such as strong acids and bases, which pose potential risks to both human health and the environment. Moreover, the raw material is subject to seasonal fluctuations and compositional variability, and the presence of residual proteins may provoke allergic reactions. Consequently, certain consumer groups, particularly individuals with seafood allergies, are advised to avoid products containing commercial chitosan [5,6].

This scenario also raises concerns regarding environmental sustainability, particularly in the context of Sustainable Development Goal 12 (SDG12-Responsible Consumption and Production), which aims to reduce the environmental impact of industrial processes and promote sustainable practices across sectors. The conventional production of chitosan, which involves harsh chemicals, contributes to pollution and waste generation, posing risks to both human health and the environment. These challenges highlight the need for more sustainable alternatives, such as fungal-derived chitosan, which can be produced with a significantly lower environmental footprint.

The extraction of chitin from fungi primarily involves demineralization and deproteinization steps, followed by deacetylation to convert chitin into chitosan. Compared to traditional sources, such as crustaceans, fungal cell walls contain lower concentrations of minerals and proteins, allowing for a milder and more straightforward extraction process. In fungi, chitin is predominantly associated with other structural polysaccharides, such as β-glucan, forming chitin–glucan complexes. These associations directly influence the physicochemical properties of the extracted biopolymer [3,5,7]. The composition and structural organization of these complexes can vary significantly depending on the fungal species, mycelial developmental stage, and cultivation conditions. These factors markedly affect both the yield and physicochemical characteristics of the final product [4].

Conversely, fungal chitosan, derived from the chitin present in fungal cell walls, has emerged as a promising and sustainable alternative. This biopolymer source provides greater consistency and sustainability in production, characterized by a more consistent composition, reduced risk of allergenic contamination, and the potential for continuous production through controlled fungal biomass cultivation [8]. Among fungi with biotechnological potential, *Agaricus bisporus* is notable due to its widespread cultivation and commercial availability across numerous countries. It represents a promising source for chitosan production because of its high chitin content in the cell wall. Nonetheless, further research is necessary to optimize extraction methods and to deepen the understanding of the structural properties of fungal chitosan [9].

Compared to commercially derived chitosan, primarily obtained from crustaceans, fungal chitosan has demonstrated superior performance in certain technological applications. Its main advantages include an improved film-forming ability, an enhanced capacity for encapsulating bioactive compounds, greater suitability for controlled release systems, superior antimicrobial properties and thermal stability [10].

Given this context, the present study aims to extract and characterize chitosan derived from *Agaricus bisporus*, comparing it with commercial chitosan in terms of production processes as well as physical, chemical, and structural properties. Advanced analytical techniques were employed to enable a comprehensive evaluation of the extracted material. This research not only underscores the potential of fungal chitosan as a sustainable alternative but also contributes to a broader understanding of its applications across diverse industrial sectors.

## 2. Materials and Physical–Chemical Characterization

### 2.1. Sample Processing

The raw material used in this study was white button mushrooms (*Agaricus bisporus*), purchased from a local vendor in the city of Ouro Preto, Minas Gerais, Brazil (latitude: 20°23′58.2″ S, longitude: 43°30′45.3″ W). After purchase, the mushrooms were transported to the Food Packaging Laboratory (LABEM) at the Federal University of Viçosa (UFV), located on the Viçosa campus and affiliated with the Department of Food Technology (DTA). The mushrooms were classified as grade 1 and medium size, and the purchase date was 9 March 2024.

During the extraction process, reagents, including hydrochloric acid (Alphatec brand, São Paulo, Brazil) and sodium hydroxide (Synth brand, São Paulo, Brazil), were employed. Washing procedures were performed using demineralized water. For comparison, commercial chitosan with a degree of deacetylation greater than 75% and a viscosity of 20–300 cpsy (1% wt. in 1% acetic acid) was purchased from Sigma.

Sample preparation followed the methodology adapted from Yen and Mau (2007) and Rodrigues and Okura (2022) [11,12]. Approximately 1.5 kg of *Agaricus bisporus* mushrooms were weighed and rinsed with deionized water. The fruiting bodies were then chopped and dried in a forced-air oven at 45–50 °C for 5 h. Subsequently, the dried samples were ground using a food processor until a fine powder was obtained.

The final moisture content of the mushrooms was 88.4 + 3.96%, as determined by AOAC 930.04 [13]. Water activity (WA) was measured at 0.4946 + 0.00206 using Aqualab equipment, and pH was recorded as 6.82 + 0.0265, in accordance with AOAC method 981.12 [14]. The mushroom powder was then sieved through a 60-mesh sieve (approximately 250 µm) to promote homogenization and ensure uniformity. Dry mass (dm) was determined using an analytical balance (AUY220, Shimadzu, Philippines), and the yield from the drying step was calculated according to Equation (1):

(1) $$Yield=\frac{Final\;dry\;mass\;obtained}{Initial\;mass\;of\;samples}\;\times \;100\%$$

The powdered material was packaged in multilayer polyethylene/nylon bags, sealed, and stored at room temperature in a desiccator containing silica gel to preserve its properties until the initiation of the chitin extraction process.

### 2.2. Chitin Extraction

Chitin isolation was performed based on a methodology adapted from Erdogan et al. (2017), Hassainia et al. (2018), and Ospina Álvarez et al. (2014) [8,15,16]. The process involved two main stages: demineralization and deproteinization. After each stage, the samples were dried in an oven (NE-7918, Ethiktechnology, São Paulo, Brazil) at 40–50 °C for approximately 10 h and weighed using an analytical balance (AUY220, Shimadzu, Metro Manila, Philippines). The weights of the samples were recorded at each stage in order to calculate the yield of chitin and chitosan, as described by Equation (1).

#### 2.2.1. Demineralization

The demineralization was carried out to eliminate mineral components from the mushroom material. Approximately 35 g of mushroom powder was weighed and transferred to a beaker. Then, 300 mL of 2 M hydrochloric acid was added, fully submerging the sample. The mixture was stirred magnetically at 200 rpm (CE-1540/A-6, Cienlab, São Paulo, Brazil) and maintained at 45 + 2 °C, for 12 h. Afterward, the samples were filtered and washed until neutral pH was reached, as measured with a pH meter (PG 1800, Gehaka, São Paulo, Brazil).

#### 2.2.2. Deproteinization

The filtrate from the previous step was treated with approximately 300 mL of 2 M NaOH solution, ensuring full submersion. The mixture was stirred magnetically at 200 rpm (CE-1540/A-6, Cielab, São Paulo, Brazil) and maintained at 80 + 2 °C for 20 h. After the reaction, samples were filtered and washed with distilled water until a neutral pH was reached, as measured using a pH meter (PG 1800, Gehaka, São Paulo, Brazil).

### 2.3. Chitin Deacetylation

To produce chitosan through alkaline treatment, the methodology described by Erdogan et al. (2017) was followed [15]. The chitin samples were treated with 300 mL of 60% NaOH at 120 + 2 °C for 4 h. The samples were then filtered and washed with distilled water until neutral pH was achieved, as measured using a pH meter (PG 1800, Gehaka, São Paulo, Brazil).

### 2.4. Characterization Analysis

#### 2.4.1. X-Ray Diffraction (illustratedXRD)

Crystallinity was determined using an X-ray diffractometer (XRD-6000, Shimadzu, Kyoto, Japan) equipped with CuKα radiation, filtered through nickel, with a wavelength of 1.542 Å. The diffraction angle (2θ) range was 5° to 50°, under controlled operating conditions of 40 kV voltage and 40 mA current. Scanning was performed at a 0.020° step size and with a 0.20 s acquisition time per step [17]. The crystallinity index (CI) was calculated according to the equation proposed by Segal et al., 1959 [18]:

(2) $$CI=\frac{\left(I_{002}-I_{am}\right)}{I_{002}}\times 100$$

In Equation (2), the relative percentage of crystallinity is defined as follows:

I_002_ = Maximum diffraction intensity;
I_am_ = Amorphous diffraction intensity.

#### 2.4.2. Scanning Electron Microscopy (SEM)

Surface morphology was assessed using scanning electron microscopy (Model TM3000, Hitachi Hi-Tech, Santa Clara, CA, USA). Samples were fixed onto conductive carbon tape. Analysis was conducted in low vacuum at 5 to 15 kV, without metallic coating, to avoid charging artifacts. Images were captured at 100×, 200×, and 600× magnification [19].

#### 2.4.3. Fourier Transform Infrared Spectroscopy (FTIR) and Degree of Deacetylation

FTIR analyses were acquired using a Nicolet 6700 spectrophotometer (Thermo Scientific, Waltham, MA, USA) over the range of 4000–600 cm^−1^, with 8 cm^−1^ resolution and 32 scans per sample, following the protocol described by Moriana et al. (2016) [20]. This technique enabled the identification of key characteristic spectral bands, facilitating the determination of molecular interactions and the identification of functional groups present in the material.

The degree of deacetylation (DD) was determined based on the methodology described by Soares et al. (2019) [21], with some adaptations, using two characteristic peaks of the chitosan at 1320 cm^−1^ and 1420 cm^−1^. The calculation was carried out in two steps. First, the degree of acetylation (DA) was obtained using Equation (3); second, the degree of deacetylation (DD) was derived from Equation (4):

(3) $$DA\left(\%\right)=\;\frac{\left(\frac{A1320}{A1420}-0.3822\right)}{0.03133}$$

DD (%) = 100% − DA (%)(4)

#### 2.4.4. Thermogravimetric Analysis (TGA)

Thermal properties were evaluated using a thermogravimetric analyzer (DTG-60, Shimadzu, Kyoto, Japan). Approximately 4 mg of each sample was heated from 25 to 600 °C at a rate of 10 °C/min under a nitrogen atmosphere with a flow rate of 50 mL/min [19,22].

#### 2.4.5. Zeta Potential

Zeta potential and dynamic light scattering (DLS) were conducted using a Zetasizer (NanoZS Malvern, UK). Approximately 1.5 mg of each sample was dispersed in 2 mL of demineralized water and sonicated with a 400 W ultrasonic probe for 10 min [23,24].

#### 2.4.6. Color Analysis

Color measurements were made using a colorimeter (Colorquest^®^ XE, HunterLab, Reston, VA, USA) in the CIELAB color space described by three coordinates: L* denotes lightness, ranging from 0 (black) to 100 (white); a* represents the red (+) to green (–) axis; and b* corresponds to the blue (–) to yellow (+) axis. The measurements were performed using illuminant D65 and a 10º observation angle. The device was calibrated with white and black color standards prior to measurements [25].

#### 2.4.7. Statistical Analysis

Chitosan yield, pH, titratable acidity, and water activity (aw) measurements were performed in triplicate, and the results were reported as mean ± standard deviation. For color analysis, one-way ANOVA and Tukey’s post hoc test (5% significance) were used with Statistica 8.0 software. The remaining analyses were explored descriptively. Results were presented in tables, graphs, and figures to highlight trends and key observations.

## 3. Results and Discussion

### 3.1. Yield

Considering the initial mass of dried *Agaricus bisporus* mushroom biomass (20 g) and the final mass obtained after chitin extraction (13.7 g), a yield of 68.35% was calculated. This value reflects the efficiency of the combined demineralization and deproteinization steps, which effectively removed non-polysaccharide components, such as minerals and proteins, from the fungal cell wall. The high yield can be attributed to the intrinsic characteristics of fungal biomass, which naturally contains lower mineral content compared to crustacean sources. These findings are consistent with those reported by Gil (2023) [26], who observed higher yields in polysaccharide extraction from fungi, primarily due to their biochemical composition, characterized by a lower mineral fraction and a higher proportion of structural polysaccharides. This feature represents a significant advantage, enabling a more efficient, cleaner, and environmentally sustainable extraction process [8].

Based on the ratio between the initial mass of *Agaricus bisporus* biomass and the final mass of extracted chitosan, an overall yield of 14.6 + 1.14% was obtained. This value reflects the overall efficiency of the entire extraction process, from biomass preparation to the final product. Although directly related to the chitosan, this yield also serves as an indirect estimate of the chitin content present in the fungal cell wall. Previous studies have shown that chitin content can vary significantly depending on the part of the mushroom analyzed. For example, Di Mario et al. (2008) [27] identified 8.5% chitin in dried *A. bisporus* mycelium, whereas Wu et al. (2004) [28] reported a higher yield of 12.44% in mushroom stipes subjected to different post-harvest conditions, which may have influenced the chitin content. In the present study, the final chitosan yield of 15.4% exceeded the average chitin values typically reported in the literature. This difference may be attributed to the specific extraction conditions employed, including sodium hydroxide (NaOH) concentration, processing temperature, and exposure time. These results highlight the potential of *A. bisporus* as a promising and viable source for fungal chitosan production.

However, certain limitations must be acknowledged. Yields may vary depending on the type of sample, preparation methods, and purification procedures. Therefore, future studies should focus not only on yield but also on evaluating the functional properties of the extracted chitosan and its potential applications in areas such as biotechnology, materials engineering, and biodegradable packaging. Such investigations could broaden the understanding of fungal chitosan’s industrial relevance and enhance the value of this sustainable biopolymer.

### 3.2. X-Ray Diffraction (XRD) Data 

**Figure 1:**
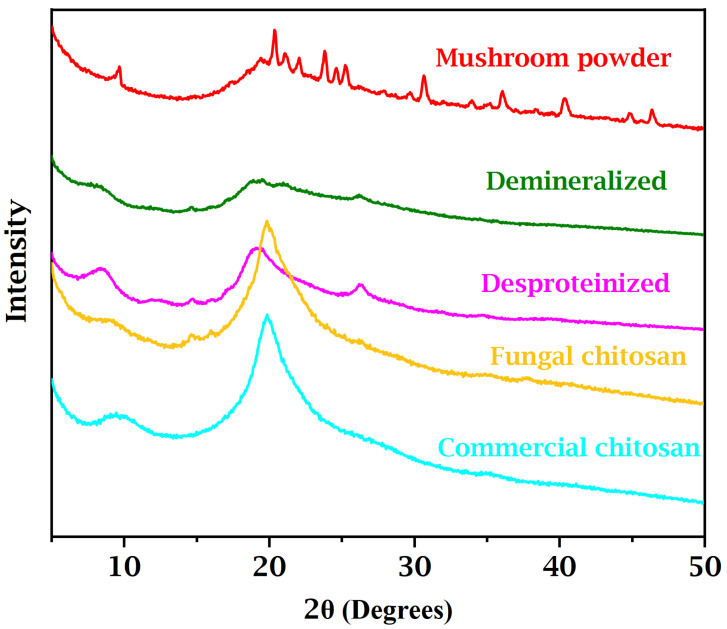
XRD patterns of mushroom powder, demineralized chitin, deproteinized chitin, and fungal and commercial chitosan.

A comparative analysis of the X-ray diffraction (XRD) patterns obtained at various stages of the chitosan extraction process from mushroom biomass reveals significant structural transformations, particularly with respect to material crystallinity (Figure 1). The initial diffractogram of raw mushroom powder exhibits well-defined peaks, notably a prominent reflection near 2θ = 10°, which is characteristic of the crystalline structure of α-chitin, along with peaks between 2θ = 21° and 2θ = 23°, associated with semicrystalline regions composed of chitin and cellulose, two major structural constituents of the fungal cell wall [29,30].

Additionally, a set of peaks observed between 2θ = 20° and 2θ = 24° can be attributed to the crystallization of mannitol, a polyol commonly found in mushrooms, whose crystalline nature is enhanced during the drying process [31]. The pattern is further complemented by peaks corresponding to inorganic minerals, such as phosphates, silicates, and oxalates, evident in the regions of 2θ = 25–27° and 2θ = 36–47°, which reflect the inherent compositional complexity of the raw fungal material.

After the demineralization step, a substantial reduction or complete disappearance of mineral-associated peaks is observed, confirming the efficiency of this treatment in removing inorganic components. Interestingly, contrary to previous reports, such as those by Poerio et al. [32] who noted an increase in crystallinity after demineralization of chitin from cicada exuviae, our findings demonstrate a notable decrease in crystallinity. This is evidenced by the attenuation of the peak near 2θ = 10° and the diminished intensity of reflections in the 2θ = 21° to 2θ = 23° range, resulting in a predominantly amorphous pattern. This behavior may be attributed to the action of hydrochloric acid used during demineralization, which not only removes mineral components but may also induce partial hydrolysis of chitin’s glycosidic bonds, thereby disrupting the crystalline lattice and reducing the overall molecular order.

Subsequently, the deproteinization step, carried out through alkaline treatment, led to further structural modifications. These changes were evident in the XRD pattern, particularly by the narrowing of the band around 2θ = 20°, which indicates a partial reorganization of the chitin chains. Although the material remained predominantly amorphous, the removal of proteins, lipids, and hemicellulosic polysaccharides achieved via solubilization and saponification reduced steric hindrance and allowed for improved molecular packing. Additionally, the presence of shoulders and secondary reflections near 2θ = 9°, 2θ = 12.5–13°, and 2θ = 23–24° suggests the persistence of characteristic α-chitin structures, even after the purification processes [33].

Finally, the deacetylation step responsible for converting chitin into chitosan resulted in XRD patterns characterized by sharper and more intense peaks, reflecting an increase in the semi-crystalline nature of the material. Both the chitosan derived from fungal biomass and the commercial standard exhibited distinct diffraction peaks, confirming the formation of organized crystalline domains. This structural reorganization is pivotal for the functional properties of chitosan, particularly in applications such as controlled drug delivery systems, wound healing, and biodegradable materials. The comparative analysis of the XRD patterns underscores the significant impact of each extraction stage on the material’s crystallinity and molecular arrangement, with deacetylation emerging as a critical step in restoring and partially stabilizing the structural order, an essential attribute for the effective biotechnological performance of chitosan [34,35].

### 3.3. Scanning Electron Microscopy Data (SEM)

**Figure 2:**
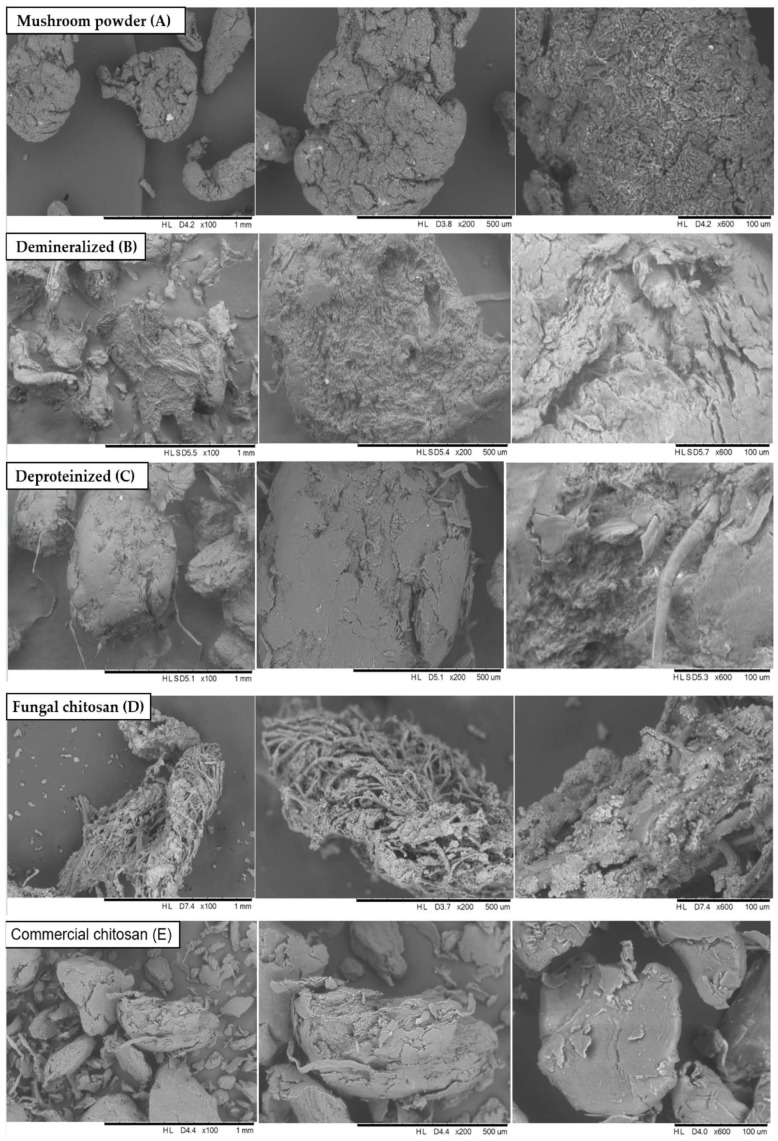
Morphology of the samples analyzed at each stage of chitosan extraction from mushroom biomass (*Agaricus bisporus*) at magnification levels of 100×, 200×, and 600×. (**A**) mushroom powder; (**B**) demineralized sample; (**C**) deproteinized sample; (**D**) fungal chitosan; and (**E**) commercial chitosan.

The raw mushroom powder exhibited a rough and heterogeneous surface morphology, characterized by a lumpy and relatively coarse morphology (Figure 2A). In contrast, the deproteinized chitin displayed a smoother and more uniform surface, indicating a certain degree of fiber alignment, molecular organization, and improved packing of the chitin chains (Figure 2C). This structural refinement supports the effective removal of interfering compounds, such as proteins, lipids, and other solubles.

The fungal chitosan (Figure 2D) revealed a fibrous and highly organized morphology, suggesting that the deacetylation step was successfully carried out. However, some surface irregularities were still observed, potentially attributed to incomplete deacetylation or the presence of residual fungal components [36,37]. Unlike crustacean-derived chitosan, which typically exhibits a more even distribution of acetyl groups along the polymer chain, fungal chitosan often exhibits clustered acetyl groups concentrated in specific regions [3].

The commercial chitosan sample exhibited the most homogeneous and well-organized morphology among all samples (Figure 2E). Its highly ordered structure reflects a tightly controlled deacetylation process, resulting in a higher degree of crystallinity. This is evidenced by the presence of small, uniform, and homogeneous particles and a stable molecular arrangement, hallmarks of extensively purified, commercial-grade chitosan [38].

Based on the SEM analysis, chitosan derived from fungal biomass exhibited a porous and irregular surface morphology, which may be advantageous for applications requiring flexibility, biodegradability, and high surface area. These structural characteristics indicate potential suitability for use in tissue engineering scaffolds, controlled drug delivery systems, and water filtration membranes, where porosity facilitates cell infiltration, regulates release kinetics, and enhances contaminant adsorption. Nonetheless, additional physicochemical and biological evaluations are necessary to validate these functional attributes. In contrast, commercial chitosan displayed a denser and homogeneous morphology, suggesting a higher degree of molecular organization. This structural uniformity is favorable for applications demanding mechanical strength and stability, such as wound dressings, biodegradable packaging, and biomedical devices, where structural integrity and durability are essential for performance.

### 3.4. FT-IR Data

**Figure 3:**
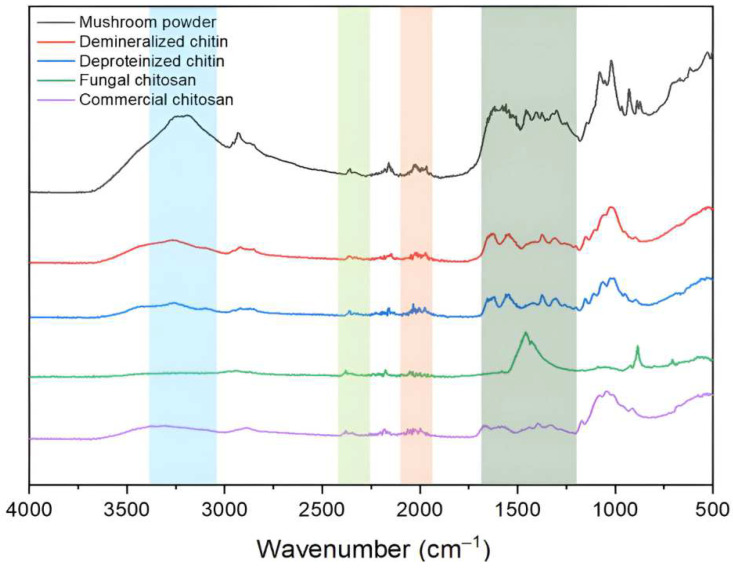
FTIR spectra of the raw mushroom powder, demineralized sample, deproteinized chitin, fungal chitosan, and commercial chitosan.

As shown in Figure 3, the FT-IR spectra of raw mushroom powder, demineralized sample, deproteinized chitin, fungal chitosan, and commercial chitosan are presented. The spectrum of the raw mushroom powder (black line) displayed broad absorption bands in the range of 3000–3500 cm^−1^ corresponding to O-H and N-H stretching vibrations, indicative of hydroxyl and amine groups associated with the chitin structure, particularly in N-acetylglucosamine units. A distinct peak was observed around 1650 cm^−1^, attributed to the amide I band (C=O stretching), a characteristic feature of the chitin. These findings are consistent with those reported by Hashem Rasti et al. (2017) [39], who identified similar FT-IR patterns in chitin extracted from the chiton shell, including sharp peaks at 627.07 cm^−1^ (out-of-plane OH), 1036.18 cm^−1^ (C-O), and 1735.26 cm^−1^ (C=O), along with a broad band between 3200 and 3500 cm^−1^ associated with N-H and O-H stretching vibrations.

It is also important to consider the potential contribution of mannitol, a sugar alcohol commonly found in mushrooms, to the FT-IR spectrum of the raw powder. Mannitol tends to crystallize during drying processes and exhibits characteristic FT-IR absorption bands that may overlap with polysaccharide signals, particularly in the regions of 3200–3500 cm^−1^ (O–H stretching), 2850–2950 cm^−1^ (C–H stretching), and 1000–1150 cm^−1^ (C–O stretching). When present in significant quantities, mannitol can influence the intensity and shape of these bands, potentially complicating the interpretation of chitin-specific features in unpurified samples. Therefore, the FT-IR spectrum of the raw mushroom powder likely reflects a complex mixture of chitin, native polysaccharides, proteins, and low-molecular-weight crystalline compounds such as mannitol [40,41,42].

Some alterations were observed in the demineralized sample (red line), notably a decrease in the intensity of the broad O–H and N–H stretching bands within the 3200–3500 cm^−1^ region. These broad bands, typically attributed to hydroxyl groups and hydrogen bonding, are generally prominent due to the abundance of –OH groups in chitin and related compounds. The observed reduction in intensity suggests a weakening of intermolecular hydrogen bonding following the removal of mineral components. The C–H stretching bands between 2850 and 2950 cm^−1^, associated with –CH_2_ and –CH groups, as well as the bending band near 1400 cm^−1^, linked to –OH bending and hydrogen bonding, remained present but showed no substantial shifts. In the 1000–1150 cm^−1^ region, multiple strong bands corresponding to C–O stretching vibrations of primary and secondary alcohols were observed, indicating the persistence of polysaccharide structures.

Although the demineralized sample remained predominantly amorphous, the peak at 1650 cm^−1^, corresponding to the C=O stretching vibration of the amide I band, appeared slightly sharper, suggesting the beginning of structural reorganization within the chitin matrix. Studies on chitin extracted from the exoskeleton of *Hermetia illucens* larvae have shown that demineralized chitin retains its characteristic FT-IR peaks, supporting the notion that mineral removal does not substantially alter chitin’s molecular structure [43]. Nevertheless, in the present study, subtle spectral variations in these key regions suggest minor conformational adjustments.

In the FT-IR spectrum of the deproteinized chitin (blue line), the band near 1550 cm^−1^, associated with the amide II vibrations (N–H bending and C–N stretching), remained evident, albeit with slightly lower intensity than in previous stages. This suggests that the alkaline treatment did not entirely remove residual proteins. The N–H stretching band around 3300 cm^−1^ also persisted, as expected, due to its fundamental presence in the chitin structure. The 1650 cm^−1^ peak (amide I) corresponding to C=O stretching appeared somewhat sharper, potentially reflecting initial molecular reorganization within the chitin chains. However, a notable reduction in the amide II band was only observed in the spectra of fungal and commercial chitosan, indicating that protein removal was more effectively accomplished during deacetylation rather than in the deproteinization step. This finding suggests that the standard alkaline deproteinization process may be insufficient for the complete elimination of protein content from the biomass.

The FT-IR spectrum of fungal chitosan (green line) showed no detectable bands near 1650 cm^−1^ (amide I) and 1550 cm^−1^ (amide II), indicating that most acetylated structures were removed during processing. However, no distinct absorption band was observed near 1590 cm^−1^, commonly attributed to the bending vibration of free amino groups (–NH_2_) and often used as a chitosan marker. This suggests that, although acetyl groups were removed, the formation or detection of free amine groups was not evident under the experimental conditions employed. A clear peak near 1450 cm^−1^ was observed, corresponding to CH_2_ bending in the polymer backbone. In contrast, commercial chitosan exhibited a weak amide I band and a pronounced peak at approximately 1590 cm^−1^, consistent with the presence of primary amines and a more complete deacetylation process. These findings imply that fungal chitosan underwent partial or structurally distinct deacetylation compared to the commercial counterpart [44,45,46].

The degree of deacetylation (DD) is a critical parameter that directly affects the physicochemical and biological properties of chitosan. In this study, DD values were determined via Fourier transform infrared spectroscopy (FTIR), calculated based on the ratio of the absorbance intensities at 1320 cm^−1^ and 1420 cm^−1^. The results for both fungal-derived and commercial chitosan samples are summarized in Table 1.

The DD of the commercial chitosan was found to be 77.27%, which is lower than the value reported by the manufacturer (DD ≥ 95%). This discrepancy may be attributed to factors such as the sensitivity of the analytical method for sampling moisture content, sample thickness, and instrumental signal variations [37]. As noted by Hosseinnejad and Jafari (2016) [47], industrial-scale extraction is performed under strictly controlled conditions, including temperature regulation, filtration, and successive washing steps, which can lead to the removal of highly deacetylated fractions. This phenomenon likely contributes to the slight reduction in DD observed in the final commercial product.

The discrepancy observed between the experimental and theoretical values underscores the importance of accurately determining the degree of deacetylation, particularly in samples subjected to multiple purification steps, as in this study. The results demonstrate that, despite slight variations in DD, the extraction process effectively induced structural modifications in chitin derived from mushrooms. Nonetheless, the methodology could be optimized to achieve higher DD values. This conversion yielded chitosan with physicochemical properties comparable to those of commercial chitosan, thereby supporting the feasibility of using alternative sources, such as fungi, for the production of high-quality chitosan [48].

### 3.5. Thermogravimetric (TGA) Data

**Figure 4:**
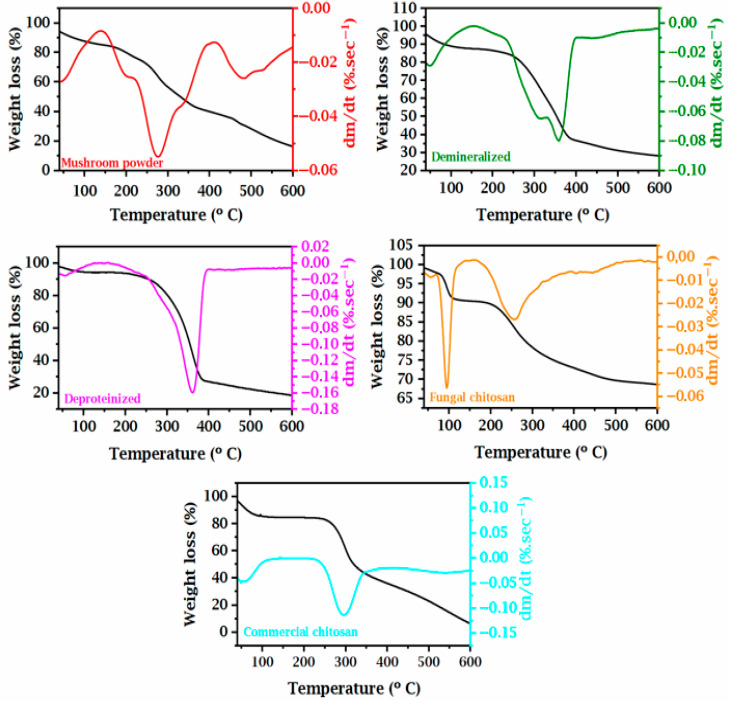
Thermogravimetric curves (TGs) and their derivatives (DTGs) for mushroom powder, demineralized chitin, deproteinized chitin, fungal chitosan, and commercial chitosan.

Thermogravimetric analysis (TGA), complemented by derivative thermogravimetric analysis (DTG), was employed to characterize the thermal stability of samples obtained at various stages of the chitosan extraction process from fungal biomass, and to compare them with commercially available chitosan. The comparison of thermal profiles revealed significant differences in thermal stability and composition among the samples, reflecting the structural changes induced by the purification steps. Figure 4 displays the TGA and DTG curves for the different samples: mushroom powder, demineralized sample, deproteinized chitin, fungal chitosan, and commercial chitosan. The weight loss (%) was plotted against temperature (°C), ranging from 25 °C to 600 °C.

The raw mushroom powder exhibited an initial mass loss of approximately 8% below 150 °C, attributed primarily to the evaporation of adsorbed water and volatile constituents. This was followed by a major degradation event between 220 °C and 350 °C, with an estimated mass loss of 55%, corresponding to the thermal decomposition of primary organic constituents, including chitin, cellulose, proteins, and non-structural polysaccharides [49,50,51]. Beyond 350 °C, a gradual residual mass loss was observed, accumulating to over 70% total mass loss by 600 °C, with the residual mass composed of mineral content and thermally stable carbonaceous compounds.

In the demineralized chitin sample, the initial mass loss up to 150 °C was decreased to approximately 6%, indicating a lower water and volatile content due to the removal of hygroscopic salts [52,53]. The primary degradation event occurred between 250 °C and 350 °C, resulting in a mass loss of around 50%, mainly attributed to the decomposition of partially purified chitin. Additionally, the residual mass above 400 °C was lower compared to that of the mushroom powder, confirming the effective removal of inorganic components during the demineralization step.

Deproteinized chitin exhibited a more distinct thermal profile, characterized by an initial mass loss of approximately 5% below 120 °C, followed by a sharp degradation event between 280 °C and 350 °C, resulting in a mass loss of about 58%. The absence of multiple degradation events in the intermediate temperature range suggests increased sample homogeneity, reflecting the effective removal of proteins, lipids, and other interfering compounds during the alkaline treatment.

The fungal chitosan sample showed an initial mass loss of 8% up to 150 °C, attributed to the elimination of residual moisture. The primary decomposition occurred between 250 °C and 350 °C, with an estimated mass loss of 55%. This earlier onset of degradation, along with the relative decrease in thermal stability compared to chitin, aligns with structural modifications caused by deacetylation, which reduce hydrogen bonding density within the polymer matrix and increase susceptibility to thermal degradation.

Commercial chitosan exhibited a thermal profile similar to that of fungal chitosan, with an initial mass loss of 3% up to 120 °C, followed by a main degradation event between 250 °C and 350 °C, resulting in approximately 50% mass loss. The lower degradation rate intensity observed in the commercial sample may be attributed to differences in the degree of deacetylation, molecular weight, or purity compared to the fungal-derived chitosan.

Overall, the comparative analysis of TGA and DTG curves indicates a progressive reduction in residual mass at elevated temperatures as the extraction and purification advanced, confirming the efficient removal of minerals and proteins. Furthermore, the primary thermal degradation event became increasingly concentrated within a narrower temperature range, a feature typical of more homogeneous and structurally refined biopolymers. Both fungal and commercial chitosan exhibit thermal stability suitable for industrial and biomedical applications, albeit lower than that of native chitin due to the loss of acetyl groups and associated molecular reordering during deacetylation.

### 3.6. Zeta Potential Data

**Figure 5:**
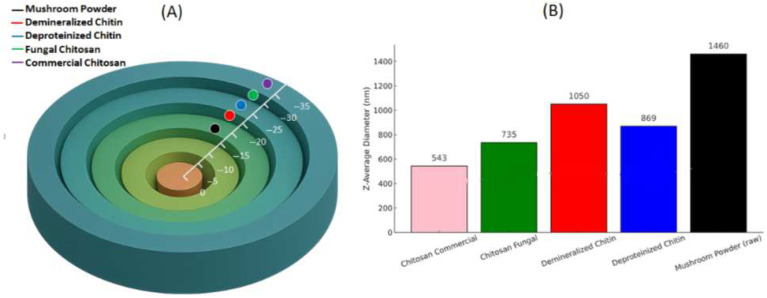
(**A**) Schematic representation of ζ-potential across different processing stages: mushroom powder, demineralized chitin, deproteinized chitin, fungal chitosan, and commercial chitosan. (**B**) Average hydrodynamic diameter (nm) of samples at each stage, illustrating a progressive decrease in particle size and improved homogeneity with purification steps.

Zeta potential analysis (ζ-potential) was conducted to evaluate the surface charge and colloidal stability of biopolymers, such as chitosan samples, in aqueous suspension (Figure 5). These measurements provide insight into the electrokinetic behavior of the particles, which plays a crucial role in determining their interactions with other molecules and surfaces. In colloidal dispersions, high absolute ζ-potential values (either positive or negative) generally indicate strong electrostatic repulsion between particles, which enhances stability and prevents aggregation [54,55].

The initial ζ-potential measured for the unprocessed mushroom powder was −18.0 ± 1.04 mV, indicating a relatively low negative surface charge. This suggests limited colloidal stability, likely attributable to the presence of impurities and the heterogeneous composition of the raw biomass. These findings are consistent with previous studies; for example, phenol-functionalized silver nanoparticles synthesized using mushroom extracts exhibited zeta potential values around −20 mV, indicative of moderate colloidal stability [56]. Likewise, investigations on chitin–glucan nanofibers derived from mushrooms have demonstrated that raw materials initially display low ζ-potential values, which increase markedly following purification [57].

Following demineralization, the absolute value of the ζ-potential increased to −24.3 ± 0.63 mV. This enhancement in negative surface charge can be attributed to the removal of mineral ions, such as Na^+^, Ca^2+^, and Mg^2+^, which reduce ionic shielding effects and improve colloidal stability. During the deproteinization step, the ζ-potential slightly decreased in magnitude to −19.96 ± 0.88 mV, possibly due to the partial adsorption of Na^+^ ions from the sodium hydroxide solution used in this process, which may have limited further increases in negative charge [58,59]. For example, a study on chitin nanofibers derived from shrimp shell waste reported a significant increase in ζ-potential after demineralization, indicating enhanced dispersion stability [60].

The conversion of chitin into chitosan through deacetylation markedly improved the surface charge characteristics, as observed in both fungal and commercial samples. Fungal-derived chitosan exhibited a ζ-potential of −27.76 ± 0.91 mV, reflecting a higher exposure of amino groups and greater purity, both of which contribute to enhanced colloidal stability. This finding is consistent with previous studies demonstrating that increased deacetylation correlates with an increase in negative surface charge, thereby improving the suspension stability of chitosan [2,61].

Commercial chitosan exhibited the highest negative ζ-potential value of −30.93 ± 0.69 mV, reflecting its higher degree of deacetylation and industrial-grade purity. These results underscore the critical role of deacetylation in modulating the electrokinetic and colloidal properties of chitosan. Increased deacetylation enhances molecular uniformity and reproducibility, key attributes for advanced functional applications, while also strengthening electrostatic repulsion between particles, thereby reducing aggregation [62].

### 3.7. Color Analysis Data

**Figure 6:**
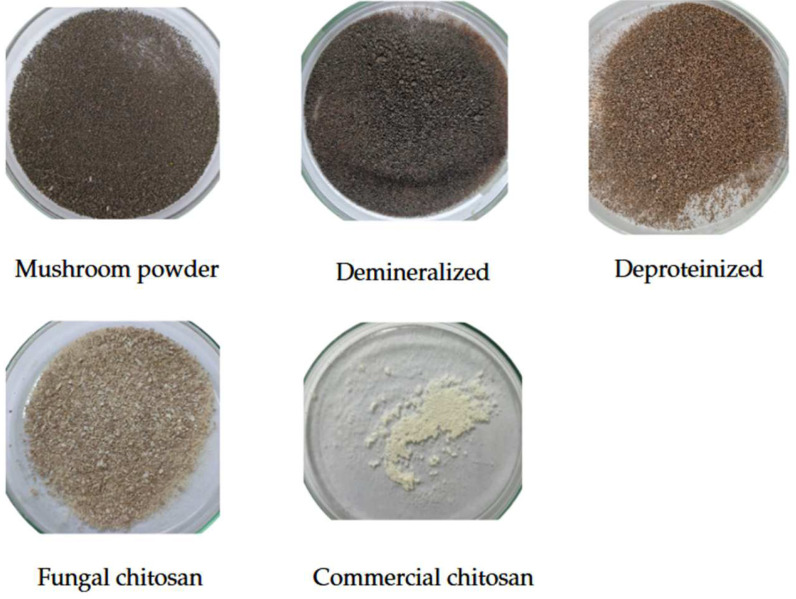
Image of samples along the process of fungal chitosan production.

The colorimetric characteristics of the samples were evaluated to assess the visual changes occurring throughout the stages of fungal chitosan extraction, as illustrated in Figure 6. The corresponding colorimetric data are summarized in Table 2.

The L* color coordinate of fungal-derived chitosan was 71.97 ± 0.44, indicating that it was significantly darker than commercial chitosan (*p* ≤ 0.05). This difference may be attributed to the presence of intrinsic fungal pigments, as the extraction process employed in this study did not include additional purification or decolorization steps. Supporting this observation, Pellis et al. (2022) [62] reported that lower L* values (L* < 30) in fungal-derived chitosans are often associated with the presence of melanin and other typical pigments found in fungi. Regarding a* and b* coordinates, we observed that the a* value was 3.35 ± 0.04, indicating a slight red tint, while the b* value of 13.03 ± 0.11 suggests a yellowish hue. The calculated chroma (C*) was 13.45 ± 0.11, reflecting a moderate color saturation. These results indicate a noticeable presence of chromophoric compounds, which are typically more prevalent in chitosans derived from marine sources [63].

The demineralized sample exhibited a low lightness (L*) value of 23.11 ± 0.02, indicating a dark coloration similar to that of the original mushroom biomass [64]. The deproteinized sample showed a slightly higher L* value of 47.92 ± 0.83, although it remained in a similar range of lightness. The a* parameter was 5.96 ± 0.12, reflecting a continued tendency toward red, while the b* value increased to 14.99 ± 0.34, suggesting a more intense yellow hue. The observed increases in both a* and b* parameters following protein removal may indicate that the deproteinization process promoted the oxidation of phenolic compounds present in the fungal matrix.

The mushroom powder exhibited a lightness (L*) value of 30.50 ± 3.36, indicating a darker coloration compared to commercial chitosan. This result suggests that the demineralization and deproteinization steps contribute to the reduction of darker color compounds. These findings are consistent with those reported by Mapari et al. (2006), who observed L* values ranging from 30 to 40 in untreated fungal raw materials [65].

Commercial chitosan exhibited the highest lightness among all evaluated samples, with an L* value of 86.90 ± 0.28 (*p* ≤ 0.05), indicating a clear and homogeneous appearance. The a* (0.87 ± 0.07) and b* (14.75 ± 0.14) values reflected a slight reddish-yellowish hue. According to Kita (2022) [66], a lighter color in chitosan is typically associated with lower impurity levels and a higher degree of deacetylation. These characteristics make commercial chitosan particularly suitable for applications where visual appearance is critical, such as in the pharmaceutical and cosmetic industries [66,67,68].

## 4. Conclusions

In this study, chitosan was successfully extracted from *Agaricus bisporus* biomass through a sequential process involving demineralization, deproteinization, and deacetylation. Comprehensive characterization techniques—including XRD, FTIR, TGA, SEM, zeta potential, and colorimetric analyses—confirmed the structural and physicochemical conversion of fungal chitin into chitosan. Compared to commercial chitosan, the fungal-derived material exhibited lower crystallinity and thermal stability but presented a more porous and fibrous morphology, favorable surface charge, and a moderate degree of deacetylation. These characteristics enhance its potential for use in biodegradable films, active packaging, and biomedical applications, where properties such as flexibility, porosity, and biocompatibility are essential. The results underscore the viability of fungal biomass as a sustainable and renewable source of chitosan, aligning with circular economy principles and offering a promising alternative to crustacean-derived biopolymers for applications in the food and healthcare industries. Future studies should focus on assessing the bioactivity of the extracted chitosan, particularly its antimicrobial and antioxidant properties, and on benchmarking its performance against commercial standards. Furthermore, optimizing extraction parameters for larger-scale production and exploring its application in food packaging, agriculture, or biomedical fields would provide a broader understanding of its potential as a biobased alternative.

## Figures and Tables

**Table 1:** Degree of acetylation and deacetylation.

Sample	DA (%)	DD (%)
Fungal Chitosan	38.43	61.57
Commercial Chitosan	22.73	77.27

**Table 2:** Color attributes of mushroom powder and chitosam samples.

Sample	L*	a*	b*
Commercial Chitosan	86.90 ± 0.28 ^a^	0.87 ± 0.07 ^c^	14.75 ± 0.14 ^a^
Fungal Chitosan	71.97 ± 0.44 ^b^	3.35 ± 0.04 ^b^	13.03 ± 0.12 ^ab^
Deproteinized Chitosan	47.92 ± 0.83 ^c^	5.96 ± 0.12 ^a^	14.99 ± 0.34 ^a^
Demineralized Chitosan	23.11 ± 0.02 ^d^	3.21 ± 0.27 ^b^	6.77 ± 0.24 ^c^
Mushroom Powder	30.50 ± 3.36 ^e^	5.36 ± 0.70 ^a^	11.36 ± 2.19 ^b^

Legend: Values are expressed as mean ± standard deviation of triplicate. Different lowercase letters within the same column indicate statistically significant differences (*p* ≤ 0.05) according to Tukey’s test for the color parameters of powdered samples.

## Data Availability

The original contributions presented in this study are included in the article. Further inquiries can be directed to the corresponding authors.

## References

[1] Batista A.C.L., Bandeira M.G.L., Souza Neto F.E.D., Paiva W.D.S., Rodrigues D.N.R., Costa A.C.A.D.A. Obtenção de Quitosana Fúngica Crescida em Meio Alternativo Constituído com Farinha de Carapaça de Camarão. https://rsc.revistas.ufcg.edu.br/index.php/rsc/article/view/309.

[2] Wang H., Roman M., Wang H., Roman M. (2023). Effects of Chitosan Molecular Weight and Degree of Deacetylation on Chitosan−Cellulose Nanocrystal Complexes and Their Formation. Molecules.

[3] Huq T., Khan A., Brown D., Dhayagude N., He Z., Ni Y. (2022). Sources, Production, and Commercial Applications of Fungal Chitosan: A Review. J. Bioresour. Bioprod..

[4] Islam N., Hoque M., Taharat S.F. (2023). Recent advances in extraction of chitin and chitosan. World J. Microbiol. Biotechnol..

[5] Alimi B.A., Pathania S., Wilson J., Duffy B., Frias J.M.C. (2023). Extraction, quantification, characterization, and application in food packaging of chitin and chitosan from mushrooms: A review. Int. J. Biol. Macromol..

[6] Rebouillat S., Pla F. (2019). A Review: On Smart Materials Based on Some Polysaccharides; within the Contextual Bigger Data, Insiders, “Improvisation” and Said Artificial Intelligence Trends. J. Biomater. Nanobiotechnol..

[7] Ozel N., Elibol M. (2024). Chitin and chitosan from mushroom (*Agaricus bisporus*) using deep eutectic solvents. Int. J. Biol. Macromol..

[8] Hassainia A., Satha H., Boufi S. (2018). Chitin from *Agaricus bisporus*: Extraction and characterization. Int. J. Biol. Macromol..

[9] Del Rocío Urbina-Salazar A., Inca-Torres A.R., Aguilar M.D.P.C., Palomas J.B. (2020). Preparación de quitina fúngica a partir de subproductos de hongos comestibles (*Agaricus bisporus*). Polo Del Conoc. Rev. Científico-Prof..

[10] Zargar V., Asghari M., Dashti A. (2015). A Review on Chitin and Chitosan Polymers: Structure, Chemistry, Solubility, Derivatives, and Applications. ChemBioEng Rev..

[11] Yen M.-T., Mau J.-L. (2007). Physico-chemical characterization of fungal chitosan from shiitake stipes. LWT—Food Sci. Technol..

[12] de Moura Rodrigues G., Okura M.H. (2022). Avalição da composição centesimal e temperatura de secagem de cogumelos Shiitake. Res. Soc. Dev..

[13] AOAC (2005). Official Method 930.04—Moisture in Dried Fruits and Vegetables. Official Methods of Analysis of the AOAC International.

[14] AOAC (2005). Official Method 981.12—pH of Fruit Products. Official Methods of Analysis of AOAC International.

[15] Erdogan S., Kaya M., Akata I. (2017). Chitin extraction and chitosan production from cell wall of two mushroom species (*Lactarius vellereus* and *Phyllophora ribis*). AIP Conf. Proc..

[16] Ospina Álvarez S.P., Ramírez Cadavid D.A., Escobar Sierra D.M., Ossa Orozco C.P., Rojas Vahos D.F., Zapata Ocampo P., Atehortúa L. (2014). Comparison of Extraction Methods of Chitin from *Ganoderma lucidum* Mushroom Obtained in Submerged Culture. BioMed Res. Int..

[17] Liao J., Huang H. (2020). A fungal chitin derived from Hericium erinaceus residue: Dissolution, gelation and characterization. Int. J. Biol. Macromol..

[18] Segal L., Creely J.J., Martin A.E., Conrad C.M. (1959). An Empirical Method for Estimating the Degree of Crystallinity of Native Cellulose Using the X-Ray Diffractometer. Text. Res. J..

[19] Kim H., Kang S., Li K., Jung D., Park K., Lee J. (2021). Preparation and characterization of various chitin-glucan complexes derived from white button mushroom using a deep eutectic solvent-based ecofriendly method. Int. J. Biol. Macromol..

[20] Moriana R., Vilaplana F., Ek M. (2016). Cellulose Nanocrystals from Forest Residues as Reinforcing Agents for Composites: A Study from Macro- to Nano-Dimensions. Carbohydr. Polym..

[21] de Souza Soares L., Perim R.B., de Alvarenga E.S., de Moura Guimarães L., de Carvalho Teixeira A.V.N., dos Reis Coimbra J.S., de Oliveira E.B. (2019). Insights on physicochemical aspects of chitosan dispersion in aqueous solutions of acetic, glycolic, propionic or lactic acid. Int. J. Biol. Macromol..

[22] Sousa M.M.D., Soares N.D.F.F., Silva D.D.J., Coimbra J.S.D.R. (2020). Desenvolvimento De Nanocompósito Biodegradável Ativo Incorporado Com Nanocristais De Celulose Modificados E Com Lisozima. Master’s Thesis.

[23] Souza Neto F.E.D. (2017). Caracterização e Avaliação do Potencial da Quitosana Extraída de Cunninghamella Elegans na Formação de Cristais de Oxalato de cálcio e Atividade Antioxidante. Master’s Thesis.

[24] Paiva W.D.S. (2017). Quitosana Fúngica na Produção de Biomaterial Membranoso Modificado por Plasma de Descarga em Barreira Dielétrica (DBD). Master’s Thesis.

[25] Espitia P.J.P., Soares N.D.F.F., Teófilo R.F., dos Reis Coimbra J.S., Vitor D.M., Batista R.A., Ferreira S.O., de Andrade N.J., Medeiros E.A.A. (2013). Physical–mechanical and antimicrobial properties of nanocomposite films with pediocin and ZnO nanoparticles. Carbohydr. Polym..

[26] Gil I.M. (2023). Cultivo Submerso do Fungo Hericium Erinaceus: Avaliação do Meio de Cultivo na Produção de Biomassa Micelial e Exopolissacarídeo. Bachelor’s Thesis.

[27] Di Mario F., Rapanà P., Tomati U., Galli E. (2008). Chitin and chitosan from Basidiomycetes. Int. J. Biol. Macromol..

[28] Wu T., Zinavovic S., Draughon F.A., Sams C.E. (2004). Chtin and Chitosan—Value-Added Products from Mushroom Waste. J. Agric. Food Chem..

[29] Delezuk J.A.D.M., Pavinatto A., Campana-Filho S.P. (2019). Influence of the process parameters on β-chitin and α-chitin extraction: Probing about the grinding and particles size. Mater. Today Proc..

[30] Wang C., Esker A.R. (2014). Nanocrystalline chitin thin films. Carbohydr. Polym..

[31] Yang Y., Liu J., Hu A., Nie T., Cheng Z., Liu W. (2022). A Critical Review on Engineering of d-Mannitol Crystals: Properties, Applications, and Polymorphic Control. Crystals.

[32] Poerio A., Petit C., Jehl J.-P., Arab-Tehrany E., Mano J.F., Cleymand F. (2020). Extraction and Physicochemical Characterization of Chitin from Cicada orni Sloughs of the South-Eastern French Mediterranean Basin. Molecules.

[33] Almeida C.F., Amorim I., Silva C.G., Lopes J.C.B., Manrique Y.A., Dias M.M. (2025). Recovery of Chitin from Agaricus bisporus Mushrooms: Influence of Extraction Parameters and Supercritical CO_2_ Treatment on Fresh Mushrooms and Production Residues. Molecules.

[34] Rasweefali M.K., Sabu S., Muhammed Azad K.S., Raseel Rahman M.K., Sunooj K.V., Sasidharan A., Anoop K.K. (2022). Influence of deproteinization and demineralization process sequences on the physicochemical and structural characteristics of chitin isolated from Deep-sea mud shrimp (*Solenocera hextii*). Adv. Biomark. Sci. Technol..

[35] Yuan Y., Chesnutt B.M., Haggard W.O., Bumgardner J.D. (2011). Deacetylation of Chitosan: Material Characterization and in vitro Evaluation via Albumin Adsorption and Pre-Osteoblastic Cell Cultures. Materials.

[36] Kanani N., Kurniawan T., Kosimaningrum W., Meliana Y., Jayanudin, Wardhono E. (2023). Ultrasonic irradiation as a mild and efficient protocol for the demineralization of chitin from shrimp shell wastes. S. Afr. J. Chem. Eng..

[37] Kumari S., Kumar Annamareddy S.H., Abanti S., Kumar Rath P. (2017). Physicochemical properties and characterization of chitosan synthesized from fish scales, crab and shrimp shells. Int. J. Biol. Macromol..

[38] Sul Y., Ezati P., Rhim J.-W. (2023). Preparation of chitosan/gelatin-based functional films integrated with carbon dots from banana peel for active packaging application. Int. J. Biol. Macromol..

[39] Rasti H., Parivar K., Baharara J., Iranshahi M., Namvar F. (2017). Chitin from the Mollusc Chiton: Extraction, Characterization and Chitosan Preparation. Iran. J. Pharm. Res..

[40] Valverde M.E., Hernández-Pérez T., Paredes-López O. (2015). Edible Mushrooms: Improving Human Health and Promoting Quality Life. Int. J. Microbiol..

[41] Al-khattawi A., Alyami H., Townsend B., Ma X., Mohammed A.R. (2014). Evidence-Based Nanoscopic and Molecular Framework for Excipient Functionality in Compressed Orally Disintegrating Tablets. PLoS ONE.

[42] Sharma V.K., Kalonia D.S. (2004). Effect of vacuum drying on protein-mannitol interactions: The physical state of mannitol and protein structure in the dried state. AAPS PharmSciTech.

[43] Triunfo M., Tafi E., Guarnieri A., Salvia R., Scieuzo C., Hahn T., Zibek S., Gagliardini A., Panariello L., Coltelli M.B. (2022). Characterization of chitin and chitosan derived from Hermetia illucens, a further step in a circular economy process. Sci. Rep..

[44] Cheng J., Zhu H., Huang J., Zhao J., Yan B., Ma S., Zhang H., Fan D. (2020). The physicochemical properties of chitosan prepared by microwave heating. Food Sci. Nutr..

[45] Kaya M., Baran T., Erdoğan S., Menteş A., Aşan Özüsağlam M., Çakmak Y.S. (2014). Physicochemical comparison of chitin and chitosan obtained from larvae and adult Colorado potato beetle (*Leptinotarsa decemlineata*). Mater. Sci. Eng. C.

[46] Kaya M., Baran T. (2015). Description of a new surface morphology for chitin extracted from wings of cockroach (*Periplaneta americana*). Int. J. Biol. Macromol..

[47] Hosseinnejad M., Jafari S.M. (2016). Evaluation of different factors affecting antimicrobial properties of chitosan. Int. J. Biol. Macromol..

[48] Li J., Pu T., Wang Z., Liu T. (2023). Thermal Behavior and Pyrolysis Kinetics of Mushroom Residue with the Introduction of Waste Plastics. Polymers.

[49] Tǎnase C., Odochian L., Balaeş T., Lisǎ G., Gherca D., Pui A. (2014). Study of thermal behaviour of some edible mushrooms. J. Therm. Anal. Calorim..

[50] Ramírez M.Á., González P., Fagundo J.R., Suarez M., Melian C., Rodríguez T., Peniche C. (2017). Chitin Preparation by Demineralizing Deproteinized Lobster Shells with CO_2_ and a Cationite. J. Renew. Mater..

[51] Alehosseini E., Tabarestani H.S., Kharazmi M.S., Jafari S.M. (2022). Physicochemical, Thermal, and Morphological Properties of Chitosan Nanoparticles Produced by Ionic Gelation. Foods.

[52] Corazzari I., Nisticò R., Turci F., Faga M.G., Franzoso F., Tabasso S., Magnacca G. (2015). Advanced physico-chemical characterization of chitosan by means of TGA coupled on-line with FTIR and GCMS: Thermal degradation and water adsorption capacity. Polym. Degrad. Stab..

[53] Zasoski R.J. (2008). Zeta potential. Encyclopedia of Earth Sciences Series.

[54] Danaei M., Dehghankhold M., Ataei S., Hasanzadeh Davarani F., Javanmard R., Dokhani A., Khorasani S., Mozafari M.R. (2018). Impact of Particle Size and Polydispersity Index on the Clinical Applications of Lipidic Nanocarrier Systems. Pharmaceutics.

[55] Mohanta Y.K., Nayak D., Biswas K., Singdevsachan S.K., Abd Allah E.F., Hashem A., Alqarawi A.A., Yadav D., Mohanta T.K. (2018). Silver Nanoparticles Synthesized Using Wild Mushroom Show Potential Antimicrobial Activities against Food Borne Pathogens. Mol. A J. Synth. Chem. Nat. Prod. Chem..

[56] Zhang M., Zhao K., Zhang K., Wang W., Xing J., Li Y. (2022). Influence of glucan on physicochemical and rheology properties of chitin nanofibers prepared from Shiitake stipes. Carbohydr. Polym..

[57] Mohd Affandy M.A., Rovina K. (2024). Characterization of chitosan derived from mushroom sources: Physicochemical, morphological, thermal analysis. Sustain. Chem. Pharm..

[58] Psarianos M., Marzban N., Ojha S., Schneider R., Schluter O.K. (2024). Functional and bioactive properties of chitosan produced from Acheta domesticus with fermentation, enzymatic and microwave-assisted extraction. Sustain. Food Technol..

[59] Boonmahitthisud A., Thongdonson K., Tanpichai S. (2023). Preparation of Chitin Nanofibers from Shrimp Shell Waste by Partial Deacetylation and Mechanical Treatment. J. Nat. Fibers.

[60] Mun S., Decker E.A., McClements D.J. (2006). Effect of molecular weight and degree of deacetylation of chitosan on the formation of oil-in-water emulsions stabilized by surfactant-chitosan membranes. J. Colloid Interface Sci..

[61] Yang H.C., Hon M.H. (2010). The effect of the degree of deacetylation of chitosan nanoparticles and its characterization and encapsulation efficiency on drug delivery. Polymer.

[62] Pellis A., Guebitz G.M., Nyanhongo G.S. (2022). Chitosan: Sources, Processing and Modification Techniques. Gels.

[63] Maciel V.B.V., Franco T.T., Yoshida C.M.P. (2012). Sistemas inteligentes de embalagens utilizando filmes de quitosana como indicador colorimétrico de temperatura. Polímeros.

[64] Junior L.B., Bertuci M.L., Fakhouri F.M., Martelli S.M. (2022). Caracterização e biodegradação de filmes de quitosana e proteína isolada de soja incorporados com nanocristais de celulose/Characterization and biodegradation of chitosan and soy protein isolate films incorporated with cellulose nanocrystals. Braz. J. Dev..

[65] Mapari S.A.S., Meyer A.S., Thrane U. (2006). Colorimetric Characterization for Comparative Analysis of Fungal Pigments and Natural Food Colorants. J. Agric. Food Chem..

[66] Kita D.M., Giovanella P., Yoshinaga T.T., Pellizzer E.P., Sette L.D. (2022). Antarctic fungi applied to textile dye bioremediation. An. Acad. Bras. Ciênc..

[67] Mohan K., Ganesan A.R., Ezhilarasi P.N., Kondamareddy K.K., Rajan D.K., Sathishkumar P., Rajarajessaran J., Conterno L. (2022). Green and eco-friendly approaches for the extraction of chitin and chitosan: A review. Carbohydr. Polym..

[68] Oladzadabbasabadi N., Nafchi A.M., Ariffin F., Wijekoon M.M.J., Al-Hassan A.A., Dheyab M.A., Ghasemlou M. (2022). Recent advances in extraction, modification, and application of chitosan in packaging industry. Carbohydr. Polym..

